# The Disorders of Primary Dentition

**Published:** 1882-06

**Authors:** 


### THE DISORDERS OF PRIMARY DENTITION.

There is no fact more generally recognized in infantile medicine than the influence exercised by dentition on the general health of children. "A fine child, until it commenced to cut its teeth," is not alone a familiar saying ; the experience of the greatest physicians have proven its truthfulness. In all the best works on infantile pathology, both ancient and modern, the influence of dentition is regarded as indisputable. But it must be admitted that this doctrine has been, perhaps, carried too far ; the best clinical observers have been obliged to protest against the assertions of many who would attribute most of the diseases from which children suffer to the influence of dentition. This protest is evident in the works of Rilliet and Barthez, in the clinical lectures of Trousseau, the treatise by Bouchut, and in Dr. West's remarkably practical book. None of these practitioners desire to exaggerate the rôle of dentition in the etiology of infantile diseases ; but its influence in this respect is not contested or denied. It is, in fact, an opinion current in medicine since the days of Hippocrates, and confirmed by Sydenham, Haller, Hunter, and the contemporary authors we have already cited. However, certain voices have been lifted against this generally received opinion. After showing that dentition has been accused of producing many disorders not referable to it, which is perfectly true, certain authors have denied that teething has any influence in the production of morbid phenomena.

M. Magitot, whose authority on any subject connected with the teeth cannot be denied, has particularly combated the commonly received opinion. In his work on the disorders attending the irruption of the teeth, published in the *Archives de Médecine*, in 1881, he seeks to prove that dentition has no part in the production of the disorders imputed to it, and that these disorders are merely coincidences. He recalls the opinions of Rosen, Andràl and Trousseau, that great caution should be used regarding the disorders of dentition, an opinion carried much further in the works of the distinguished English dentist, Dr. Tomes. We will consider, later on, the arguments brought forward by M. Magitot to sustain his opinions. To commence with, it seems to me useful to consider the question under three principal heads :

1st. Should we admit, in certain cases, the existence of a difficult, laborious dentition, and if so, what are the characteristic signs ?

2d. Can this difficult dentition induce disorders proper to this condition, and characterized by their nature, course and duration ?

3d. When a child is teething should all the maladies which may present themselves be laid to the influence of this physiological act ?

These different questions do not appear to me of difficult solution. Dentition is a physiological act, but not necessarily accomplished, on that account, without pathological reaction.

Children who pass through the period of primary dentition without presenting any symptoms of suffering constitute veritable exceptions.

The incisors and the first molars often pierce the gums without provoking any very marked reaction, but the irruption of the second molars, and particularly of the canine teeth, generally causes much more trouble. The phenomena which accompany the eruption of the teeth have, with reason, been divided into local and general. The most common local phenomenon is salivation, and this is the more remarkable as the buccal mucous membrane in young infants is generally dry, as Dr. West has rightly observed. At the moment when the gums become swollen and the crown of the tooth becomes prominent through the thinned surface of the gum, then the infant constantly drivels and wets several handkerchiefs in a few hours, which does not happen with a child before the fifth month. This exaggerated secretion of saliva can only be explained by the excitation transmitted to the salivary glands along the mucous membrane of their ducts.

If the irritation is more marked, and the child very impressionable, the hands are carried incessantly to the mouth, and the infant bites at and rubs against the gums any hard object within reach, particularly objects which communicate a sensation of cold. At a more advanced stage of irritation aphthæ are developed and thrush may supervene. The gravest manifestations of the irritation produced by dentition constitute what has been called odontitis infantum, characterized by a veritable stomatitis with ulcerations, which may persist so long, and determine so much suffering as to give rise to great anxiety for the life of the child, although West has never observed any case where these disorders actually caused death. In the presence of such symptoms it is difficult to deny the possibility of local disorders induced by dentition. We admit that these grave symptoms are rarely observed, but Trousseau, Guersant, West, Rilliet and Barthez mention them, and their occurrence cannot be placed in doubt. It is not, however, on this point that the principal objections have been formulated ; but against the disorders affecting the general system and imputed to dentition. It is, in effect, impossible to deny, with any appearance of reason, the existence and etiology of the local disorders, with which every physician is acquainted; but when we are in presence of disorders, such as diarrhœa, skin eruptions, convulsions, fever, etc., which present nothing special, then it is less difficult to deny the influence of dentition, and pretend that the occurrence of these symptoms at this period is a mere coincidence ; that these may be observed at any period of infancy, and that they are attributed, without any sufficient proof, to the effect on the general system of the process of teething. Nevertheless, no physician who comes frequently in contact with sick children hesitates in recognizing these evidences of constitutional disturbance as due, or referable to dentition, not in all cases, but in certain conditions of daily observation.

Reflex sympathetic phenomena are manifested in infancy with peculiar energy, of which we have daily proof ; agitation, fever, vomiting and convulsions, are often induced by slight, but constant sources of irritation, such as a badly placed pin or a boil irritated by the contact of dry and hardened dressings. How, then, can it be denied that a continuous pain in a swollen gum, which persists and cannot be relieved, may throw the infant into a condition often exceedingly distressing ?

Since the application of a blister or sinapism often determines convulsions in a nervous child, why should the continuous irritation of dentition be incapable of producing the same symptoms ?

It is true our opponents seek to establish that this pretended pain of dentition is purely mythical ; that there is no reason why the eruption of the tooth should cause pain, since there is neither effraction nor traumatism, but a very gradual wearing away or absorption of the tissues of the gum. It would then be necessary to prove and demonstrate that the different tissues pushed outward and compressed during the evolution of the tooth do not suffer in any way from this compression, which it would appear exceedingly difficult to admit.

When a vigorous child of from six to eight months, nursed by a healthy mother with excellent milk, is suddenly observed to lose sleep and gayety, become irritable and quit the breast after a few attempts to nurse, while at the same time it drivels incessantly ; when, again, after a careful examination of all the functions, no explanation can be found for this change, except that the gum is hot and painful when touched, it must necessarily be admitted that the process of dentition has caused these disorders. A few days pass, the general malaise persists, or is augmented in intensity ; diarrhœa may come on without any change in the habitual diet of the child. No active treatment is instituted, and yet, all of a sudden, the morbid symptoms disappear, and examination of the gum shows that the irruption of the tooth is complete. Can the conclusion, then, be other than that we have already formulated ?

This is not a case invented for the occasion, but a fact of daily observation ; with some children even the irruption of each tooth or group of teeth is attended by these morbid symptoms.

If these facts are not constantly observed, they are at least common, and it is certainly very rare to find a child who has not suffered more or less during the period of dentition. I have observed, for my part, a child who never had one of his first twenty teeth without suffering from one or several convulsions, and who has never presented any since the period of dentition.

What we have said of the fever, diarrhœa, and nervous disorders, can be repeated with equal justice for the multiform eruptions frequently observed in similar cases. There is nothing special to distinguish these eruptions, any more than the intestinal or nervous disorders, from those produced by other and widely different causes. They demonstrate, however, the existence of disorders in the functions of the various organs, and in the secretions, produced by dentition.

What especially characterizes these eruptions is the period of their appearance and the manner in which they follow " the evolution of the teeth, preceding and accompanying the irruption of a group of teeth and then disappearing.

If all the morbid symptoms observed during dentition cannot be ascribed to this physiological act, it is not the less true that these disorders, appearing only at this period, accompanying the irruption of the teeth and ceasing when this is accomplished, do not leave in doubt their veritable causation.

It may be admitted that dentition is not an isolated phenomenon ; that it coincides with a period of peculiar activity in the development of the child, principally with the formation of the bones, the increase of the glandular system of the digestive apparatus, and that at this moment the nervous sensibility is at its highest pitch.

This is all true, but it is certain that dentition, particularly when painful, has a preponderating rôle in the causation of these morbid manifestations.

After denying that teething can be of itself painful, M. Magitot demonstrates that traumatisms practised on young animals at the period of dentition, and affecting more or less deeply the gum or the tooth, do not determine anything comparable to the disorders attributed in children to the evolution of the teeth.

We are of opinion that there can be no analogy between the sections, punctures and tearing of the alveoli in these experiments, and the normal evolution of the tooth, which separates, forces out and slowly compresses the tissues. This last is a purely vital act, not in any manner reproduced in the traumatisms already mentioned, which induce widely different reactions.

In the same way the numerous diseases of the teeth, caries, ulcerations, etc., may often induce very severe pain without provoking any symptom resembling those observed during the period of the evolution of the teeth. The reactions are peculiar to this period and are analogous to those observed during the evolution of other organs.

The opinions of M. Magitot are reproduced in the able and conscientious thesis presented by M. Levèque to the Paris Faculty.

We have particularly considered the seven observations given by M. Levèque, and we find that several might be given as types of the disorders induced by dentition, and the interpretation of them given by the author is, to say the least, extremely far-fetched. But these objections do not mar the value of the work ; it is possible that the author will persist in his ideas if he practises dentistry exclusively. But it would be entirely different if he is called upon to follow, in their maladies and indispositions, young infants, to have them under his care during their development, and be obliged to interpret their sufferings. He would then remark, with all clinical observers, that dentition exercises a very marked influence in the production of infantile disorders, and he would soon recognize how solidly the opinion he combats to-day is established.
—*M. Blachez in Med. and Surg. Reporter.*

